# Ag Decoration and SnO_2_ Coupling Modified Anatase/Rutile Mixed Crystal TiO_2_ Composite Photocatalyst for Enhancement of Photocatalytic Degradation towards Tetracycline Hydrochloride

**DOI:** 10.3390/nano12050873

**Published:** 2022-03-06

**Authors:** Mao Tang, Yangwen Xia, Daixiong Yang, Shiji Lu, Xiaodong Zhu, Renyong Tang, Wanming Zhang

**Affiliations:** 1 School of Mechanical Engineering, Chengdu University, Chengdu 610106, China; tangmao@cdu.edu.cn (M.T.); x1278704108@163.com (Y.X.); yangdaixiong1998@163.com (D.Y.); l3221668248@163.com (S.L.); 2 Sichuan Province Engineering Technology Research Center of Powder Metallurgy, Chengdu 610106, China; 3 School of Food and Biological Engineering, Chengdu University, Chengdu 610106, China; tangrenyong@cdu.edu.cn; 4 School of Resources and Environment, Xichang University, Xichang 615000, China

**Keywords:** anatase/rutile mixed crystal TiO_2_, Ag decoration, SnO_2_ coupling, photocatalytic activity, tetracycline hydrochloride

## Abstract

The anatase/rutile mixed crystal TiO_2_ was prepared and modified with Ag decoration and SnO_2_ coupling to construct a Ag@SnO_2_/anatase/rutile composite photocatalytic material. The crystal structure, morphology, element valence, optical properties and surface area were characterized, and the effects of Ag decoration and SnO_2_ coupling on the structure and photocatalytic properties of TiO_2_ were studied. Ag decoration and SnO_2_ coupling are beneficial to reduce the recombination of photogenerated electrons and holes. When the two modification are combined, a synergistic effect is produced in suppressing the photogenerated charge recombination, making Ag@SnO_2_/TiO_2_ exhibits the highest quantum utilization. After 30 min of illumination, the degradation degree of tetracycline hydrochloride (TC) by pure TiO_2_ increased from 63.3% to 83.1% with Ag@SnO_2_/TiO_2_.

## 1. Introduction

Antibiotics have been widely used in our daily life for decades. If they are not treated as non-toxic, these antibiotics will eventually enter the water and soil, threatening human health [1,2,3,4]. Employing photocatalytic technology to degrade antibiotics is a green and efficient route, which shows broad application prospects. Among various photocatalytic materials, TiO_2_-based photocatalysts have received the most attention in the degradation of antibiotics [5,6,7].

However, due to the low utilization of sunlight and the high recombination rate of photogenerated electrons and holes of pure TiO_2_, it is necessary to modify its energy band structure and improve the visible light utilization and quantum efficiency to increase the photocatalytic activity [8,9,10]. When TiO_2_ particles contact with precious metal, forming heterojunctions, the photogenerated electrons on the conduction band of TiO_2_ will migrate to the precious metal particles until the Fermi energy level is equal, which enhances the separation photogenerated charges [11,12,13]. In the research of noble metal decoration, Ag has attracted extensive attention because of its relatively cheap and excellent modification effects [14,15,16,17,18].

Li et al. [15] fabricated Ag nanoparticle-decorated porous TiO_2_ foams using the wet-impregnation method. Due to the surface plasmon resonance (SPR) effect of Ag nanoparticles, the absorption in visible light was greatly enhanced, and thus the photocatalytic performance of TiO_2_ was improved. In other modification methods, TiO_2_ coupling with else semiconductors promotes the migration of photogenerated charges at the two-phase interface, thus, increasing the quantum utilization and photocatalytic efficiency [19,20]. SnO_2_/TiO_2_ semiconductor systems have been widely studied as the band position of SnO_2_ matches TiO_2_.

When TiO_2_ is illuminated, photogenerated holes and photogenerated electrons are generated in the valence band and conduction band, respectively. The electrons will transfer to the conduction band of SnO_2_, and the holes in the SnO_2_ valence band will migrate to TiO_2_ valence band, which separates the photogenerated electrons and holes effectively. Therefore, SnO_2_/TiO_2_ exhibits better photocatalytic performance compared with pure TiO_2_ [21,22,23]. Xun et al. introduced SnO_2_ into TiO_2_ nanotube materials to improve the quantum efficiency and obtained a higher photocatalytic activity [22].

In addition to coupling with other semiconductor materials, TiO_2_ with different crystal structures to form mix crystal heterojunctions is also conducive to the transfer of photogenerated charges, showing higher photocatalytic activity than a single crystal structure [24,25,26]. Based on these advantages, anatase/rutile mixed crystal TiO_2_ was prepared by the sol-gel method and modified by Ag decoration and SnO_2_ coupling to fabricate Ag@SnO_2_/anatase/rutile composite photocatalyst in the present work.

The crystal structure, morphology, element valence, optical properties and surface area were characterized. The photocatalytic performance of the prepared photocatalysts was assessed by taking tetracycline hydrochloride aqueous solution as the target pollutant. The effects of Ag decoration and SnO_2_ coupling on the structure and photocatalytic performance of mixed crystal TiO_2_ were investigated in detail.

## 2. Experimental Section

### 2.1. Material Preparation

Butyl titanate (Analytical Reagent, AR), absolute ethanol (AR), hydrochloric acid (AR), tin tetrachloride pentahydrate (AR), silver nitrate (AR), tetracycline hydrochloride (AR), benzoquinone (AR), ammonium oxalate (AR) and isopropanol (AR) were purchased from Chengdu Chron Chemicals Co., Ltd., (Chengdu, China). 

We added 20 mL butyl titanate and 45 mL absolute ethanol to a beaker to form mixture A. We mixed 10 mL deionized water, 15 mL absolute ethanol and 2 mL hydrochloric acid to obtained mixture B, which was added into mixture A dropwise to form a gel. After aging and drying, the powder was calcined at 550 °C for 1 h to prepare pure TiO_2_.

Silver nitrate (AgNO_3_) or tin tetrachloride pentahydrate (SnCl_4_·5H_2_O) was added into mixture B. Keep the other steps unchanged to fabricate Ag decorated TiO_2_ and SnO_2_ coupled TiO_2_, which are labelled as Ag@TiO_2_ and SnO_2_/TiO_2_. The molar ratios of Ag:Ti and Sn:Ti were 1:100 and 75:100, separately. When AgNO_3_ and SnCl_4_·5H_2_O were added simultaneously, the Ag@SnO_2_/TiO_2_ composite photocatalyst was obtained.

### 2.2. Characterization

The crystal structure was analyzed using a DX-2700 X-ray diffractometer (Dandong Haoyuan Instrument Co. Ltd., Dandong, China, XRD). The test voltage was 40 kV, the current was 30 mA, and the scanning angle was 20°–70° with the scanning speed 0.06° /s. The morphology (SEM and TEM) was observed using a FEI-Inspect F50 scanning electron microscope and a FEI-Tecnai G2 F20 transmission electron microscope (FEI Company, Hillsboro, OR, USA). Adopting an XSAM800 multifunctional surface analysis system to study the element composition and valence state (XSAM800, Kratos Ltd., Manchester, Britain, XPS). The recombination of photo-induced charges was analyzed by a F-4600 fluorescence spectrometer (F–4600, Shimadzu Group Company, Kyoto, Japan, PL). The optical absorption was tested by a UV-3600 UV-Vis spectrophotometer (UV–3600, Shimadzu Group Company, Kyoto, Japan, DRS). The BET specific surface area was analyzed by a Mike ASAP2460 analyzer (Mike Instrument Company, Atlanta, GA, USA).

### 2.3. Photocatalysis Experiment

Tetracycline hydrochloride (TC) was employed as the target pollutant to evaluate the photocatalytic performance. We added 100 mL (30 mg/L) TC aqueous solution and 0.1 g sample to a beaker and stirred for 30 min in dark. Then, a 250 W xenon lamp (Solar-350, Beijing NBeT Technology Co. Ltd., Beijing, China) was turned on as the light source for irradiation. The mixture was taken every 10 min and extracted after centrifugation, and the absorbance (A) of the supernatant at 355 nm was measured by an ultraviolet visible spectrophotometer. The degradation degree was computed by the formula as follows:

(A_0_ − A_t_)/A_0_ × 100% (1)

## 3. Results and Discussion

### 3.1. XRD Analysis

**Figure 1:**
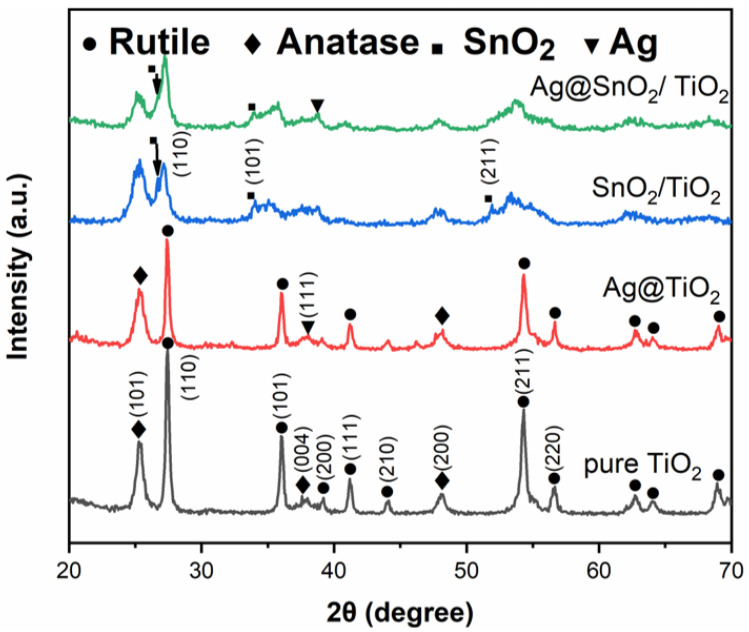
XRD patterns of pure TiO_2_, Ag@TiO_2_, SnO_2_/TiO_2_ and Ag@SnO_2_/TiO_2_.

The XRD patterns of samples are shown in Figure 1. In the pattern of pure TiO_2_, the peaks at 25.3°, 37.9° and 48.1° correspond to the (101), (004) and (200) crystal planes of anatase, and the peaks at 27.4°, 36.1° and 54.3° are indexed to the (110), (101) and (211) crystal planes of rutile, indicating that pure TiO_2_ consists of anatase and rutile and shows mixed crystal structure [24,26,27,28]. The mass fractions of anatase and rutile are 23.8% and 76.2%, respectively.

In the pattern of Ag@TiO_2_, in addition to the anatase and rutile peaks, the peak appearing at 38.1° corresponds to the (111) crystal plane of elemental Ag [29], which implies that Ag@TiO_2_ heterojunctions are formed. The peaks at 26.7°, 34.0° and 51.9° in the patterns of SnO_2_/TiO_2_ are indexed to the (110), (101), (211) crystal planes of SnO_2_ [30], showing that SnO_2_/TiO_2_ semiconductor composites are generated. The diffraction peaks of SnO_2_ and Ag appear in the pattern of Ag@SnO_2_/TiO_2_, which shows that the Ag@SnO_2_/TiO_2_ composite photocatalyst was constructed through Ag decoration and SnO_2_ coupling.

### 3.2. SEM and TEM Analyses

**Figure 2:**
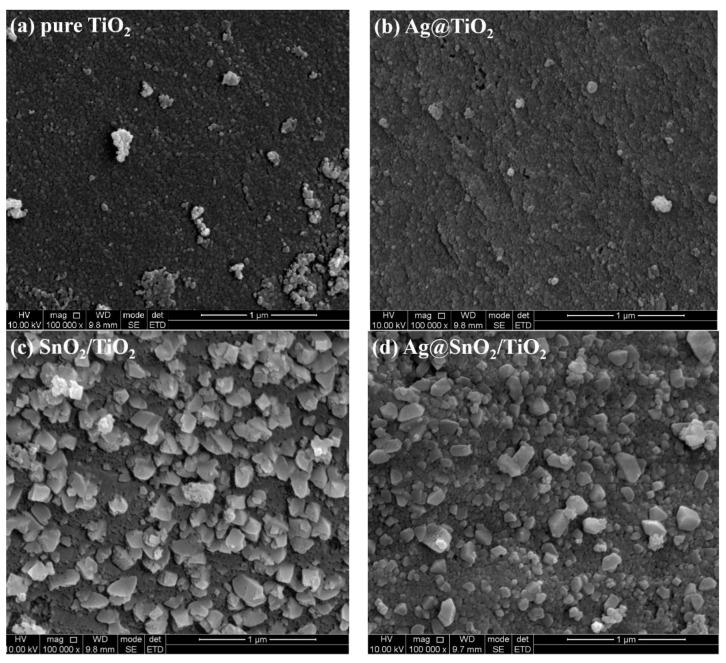
SEM images of pure TiO_2_ (**a**), Ag@TiO_2_ (**b**), SnO_2_/TiO_2_ (**c**) and Ag@SnO_2_/TiO_2_ (**d**).

Figure 2 depicts the SEM images of pure TiO_2_, Ag@TiO_2_, SnO_2_/TiO_2_ and Ag@SnO_2_/TiO_2_. Pure TiO_2_ presents a granular morphology with several agglomerates. Ag@TiO_2_ shows a similar morphology to pure TiO_2_. The particle size increases in SnO_2_/TiO_2_ and Ag@SnO_2_/TiO_2_ samples. 

**Figure 3:**
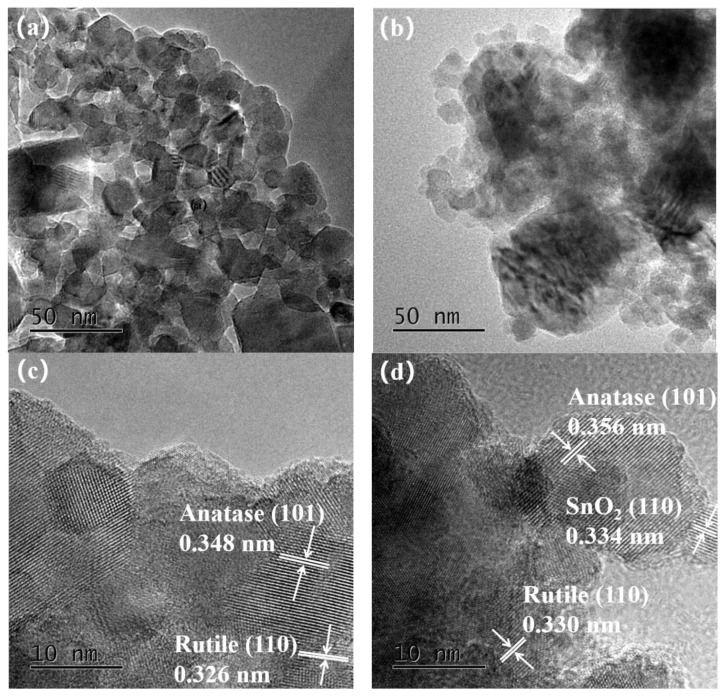
TEM and HRTEM images of pure TiO_2_ (**a**,**c**) and Ag@SnO_2_/TiO_2_ (**b**,**d**).

Figure 3 presents the TEM and HRTEM images of pure TiO_2_ (Figure 3a,c) and Ag@SnO_2_/TiO_2_ (Figure 3b,d). It is observed in Figure 3a that the particle size of pure TiO_2_ is about 20–30 nm. In Figure 3c, the marked interplanar spacing 0.348 nm corresponds to the anatase (101) crystal plane, and 0.326 nm corresponds to the rutile (110) crystal plane [7,31,32], indicating that pure TiO_2_ is composed of anatase and rutile and exhibits a mixed crystal structure. The particle size in Figure 3b is slightly smaller than pure TiO_2_, which is in the range of 15–20 nm.

The interplanar spacing 0.356 nm in Figure 3d corresponds to the anatase (101) crystal plane, which is larger than that of pure TiO_2_ (0.348 nm). As the radius of Sn^4+^ (0.0690 nm) is larger than that of Ti^4+^ (0.0605 nm), the replacement of Ti^4+^ by Sn^4+^ will cause a lattice expansion, resulting in the increase of interplanar spacing [33]. The marked interplanar spacing 0.330 nm corresponds to the rutile (110) crystal plane [34]. The interplanar spacing 0.334 nm can be attributed to the (110) crystal plane of SnO_2_ [35].

### 3.3. XPS Analysis

**Figure 4:**
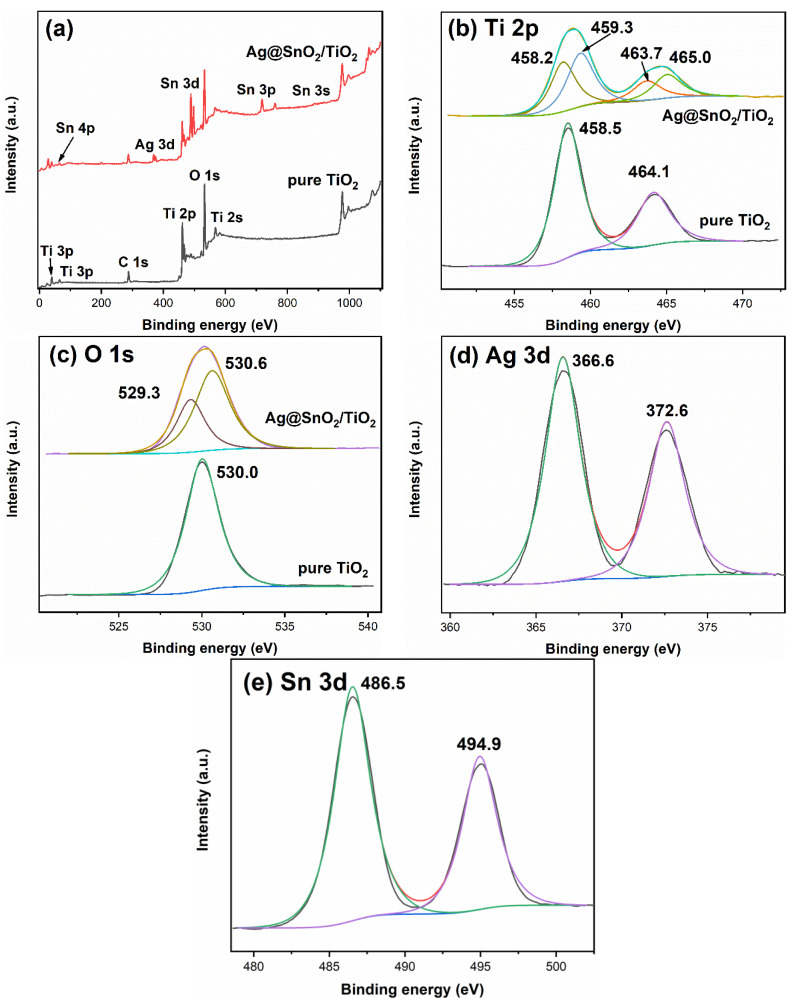
XPS spectra of pure TiO_2_ and Ag@SnO_2_/TiO_2_: total spectra (**a**), Ti 2p (**b**), O 1s (**c**), Ag 3d (**d**) and Sn 3d (**e**).

The XPS results of pure TiO_2_ and Ag@SnO_2_/TiO_2_ are shown in Figure 4. Figure 4a shows the full spectra. The constituent elements of Ag@SnO_2_/TiO_2_ are Ti, O, Sn, Ag and C. The signal of C element mainly comes from the pollution during the test. Figure 4b is the high-resolution spectra of Ti 2p. The binding energies of Ti^4+^ 2p_3/2_ and Ti^4+^ 2p_1/2_ are 458.5 and 464.1 eV, indicating that Ti element is +4 valence in pure TiO_2_ [36]. The Ti^4+^ 2p_3/2_ and Ti^4+^ 2p_1/2_ binding energies of Ag@SnO_2_/TiO_2_ shifts to higher binding energies, which are at 459.3 and 465.0 eV.

In addition, characteristic peaks at 458.2 and 463.7 eV corresponding to Ti^3+^ 2p_1/2_ and Ti^3+^ 2p_3/2_ appear, indicating that Ti exists as +3 and +4 in Ag@SnO_2_/TiO_2_ [37]. Figure 4c shows the O 1s high-resolution spectra. Pure TiO_2_ shows a characteristic peak at 530.0 eV, which corresponds to lattice oxygen (O^2−^), and the surface hydroxyl (OH^−^) peak is not obvious. The peaks of Ag@SnO_2_/TiO_2_ at 529.3 and 530.6 eV correspond to lattice oxygen (O^2−^) and surface hydroxyl (OH^−^) [34,36]. It can be observed from Figure 4d that Ag 3d shows two characteristic peaks at 366.6 and 372.6 eV corresponding to Ag 3d_5/2_ and Ag 3d_3/2_, respectively, indicating that the Ag element is in a zero-valent state [38,39]. Sn 3d (Figure 4e) exhibits two characteristic peaks at 486.5 and 494.9 eV, corresponding to Sn 3d_5/2_ and Sn 3d_3/2_, respectively, implying that Sn element is in a +4 state [34].

### 3.4. PL Analysis

**Figure 5:**
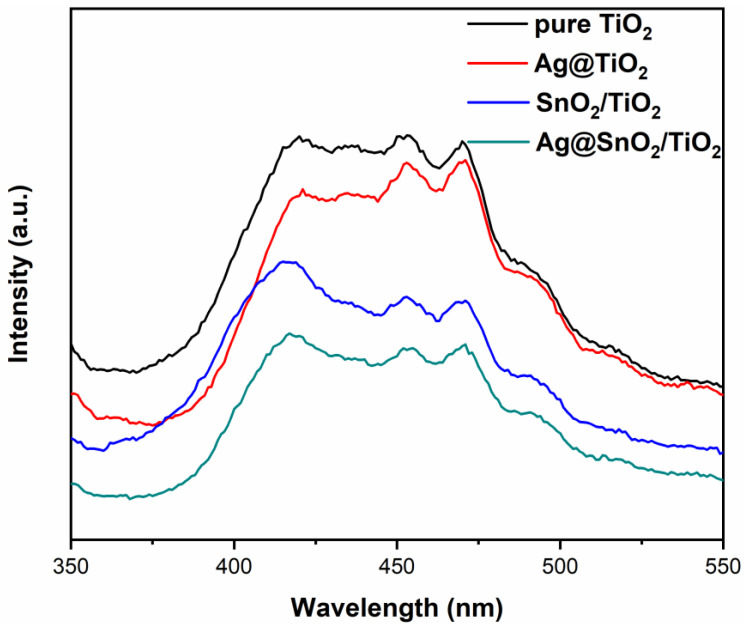
PL spectra of pure TiO_2_, Ag@TiO_2_, SnO_2_/TiO_2_ and Ag@SnO_2_/TiO_2_.

When photo-induced electron-hole pairs recombine, their excess energy will be released in the form of light, which can be responsible for the photoluminescence spectra [40,41]. Figure 5 shows the PL spectra of samples. The PL peak intensity of Ag@TiO_2_ is lower than that of pure TiO_2_, indicating that Ag decoration inhibits the recombination of photogenerated electrons and holes. As the Fermi energy level of Ag particles is lower than the conduction band position of TiO_2_, the photogenerated electrons on the conduction band of TiO_2_ will migrate to the surface of Ag particles, reducing the probability of recombination with holes in the valence band [42].

SnO_2_/TiO_2_ shows less PL peak intensity compared with pure TiO_2_, which implies that the recombination of photogenerated charges is suppressed by SnO_2_ coupling. The electrons and holes transfer rapidly at the two-phase interface, which favors the photogenerated charge separation [43]. Remarkably, the PL peak intensity of Ag@SnO_2_/TiO_2_ is the lowest, which means that Ag decoration and SnO_2_ coupling produce a synergistic effect advancing the photogenerated charge separation. After Ag decoration and SnO_2_ coupling modification, the utilization of photogenerated charges is enhanced significantly, which is beneficial to the photocatalytic activity [44].

### 3.5. DRS Analysis

**Figure 6:**
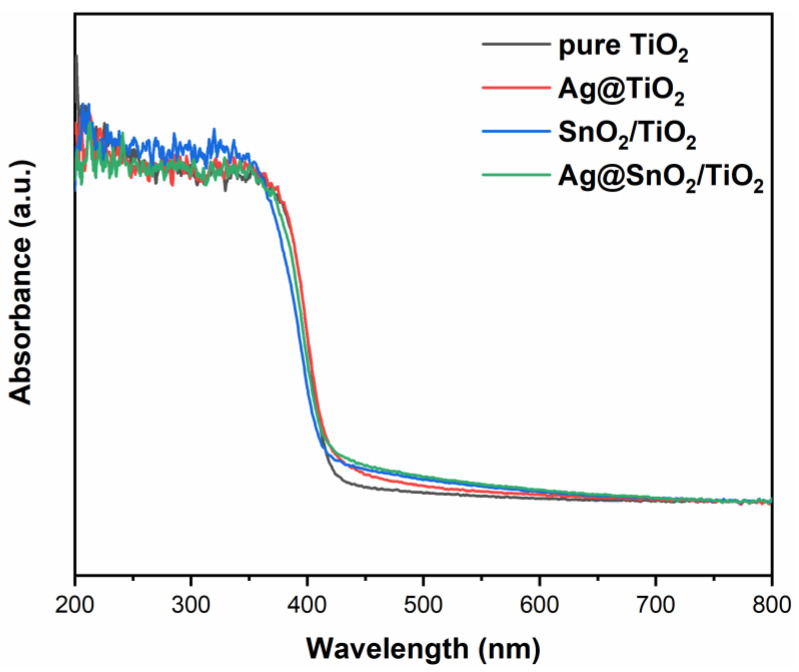
DRS spectra of pure TiO_2_, Ag@TiO_2_, SnO_2_/TiO_2_ and Ag@SnO_2_/TiO_2_.

The UV-visible absorption spectra of samples are shown in Figure 6. All samples show strong absorption in the ultraviolet region. After Ag decoration and SnO_2_ coupling, the absorption edge does not change, indicating that the modification by Ag and SnO_2_ has little effect on the band gap of TiO_2_. The absorption merely slightly increases in the 400–600 nm region after modification.

### 3.6. BET Analysis

**Figure 7:**
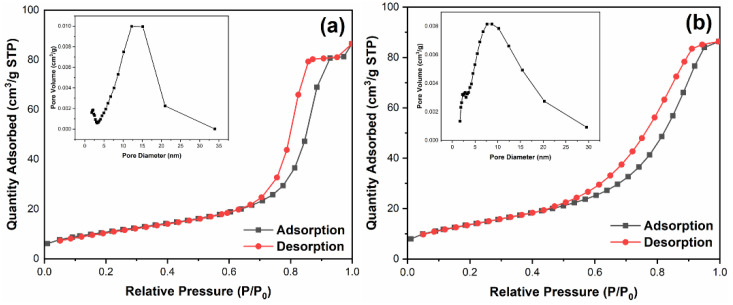
Nitrogen adsorption–desorption isotherms and the corresponding pore size distribution curves of pure TiO_2_ (**a**) and Ag@SnO_2_/TiO_2_ (**b**).

Figure 7 shows the nitrogen adsorption-desorption isotherms and their corresponding pore size distribution curves of pure TiO_2_ (a) and Ag@SnO_2_/TiO_2_ (b). Both the two samples display type IV adsorption isotherm, showing mesoporous structure [45,46]. The pore size distribution of pure TiO_2_ is between 5 and 20 nm, and the specific surface area is 39.7 m^2^/g. The pore size distribution of Ag@SnO_2_/TiO_2_ is between 5 and 30 nm, and the specific surface area is 50.5 m^2^/g.

It can be seen from XRD that, after the addition of Ag or SnO_2_, the peak intensity decreases, and the half height width increases, indicating that the grain size is reduced, which is beneficial to obtain a higher specific surface area. [47,48,49]. The increased BET surface area indicates that Ag decoration and SnO_2_ coupling modification enhances the specific surface area of anatase/rutile mixed crystal TiO_2_, which contributes to more reactive sites and higher photocatalytic efficiency [50].

### 3.7. Photocatalytic Activity

**Figure 8:**
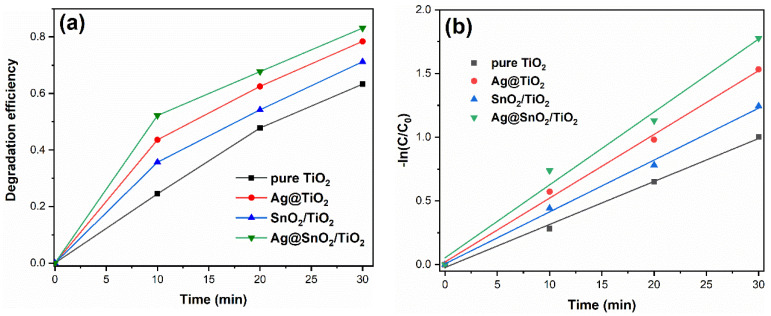
Degradation curves (**a**) and the kinetic curves of samples (**b**).

The photocatalytic performance of samples is evaluated by the degradation of TC, and the results are shown in Figure 8. After 30 min of reaction, the degradation degree of pure TiO_2_ is 63.3%. The degradation degrees are improved by modification. The degradation degrees of Ag@TiO_2_, SnO_2_/TiO_2_ and Ag@SnO_2_/TiO_2_ are 78.4, 71.2 and 83.1%, respectively. Ag@SnO_2_/TiO_2_ shows the highest photocatalytic activity. The kinetic curves of photocatalytic degradation towards TC are shown in Figure 8b. It can be observed that time t and -ln (C/C_0_) presents a linear relationship, and the photolysis reaction conforms to the first-order reaction [14]. The reaction rate constants of pure TiO_2_, Ag@TiO_2_, SnO_2_/TiO_2_ and Ag@SnO_2_/TiO_2_ are 0.034, 0.050, 0.041 and 0.057 min^−1^, respectively.

Ag decoration and SnO_2_ coupling are beneficial to the separation of photogenerated charges and quantum efficiency, which has been proven by PL spectra. When Ag decoration and SnO_2_ coupling are implemented simultaneously, a synergistic effect on suppressing the recombination of photogenerated electrons and holes is yielded. Therefore, Ag@SnO_2_/TiO_2_ shows the lowest PL peak intensity, exhibiting the highest photocatalytic activity.

**Figure 9:**
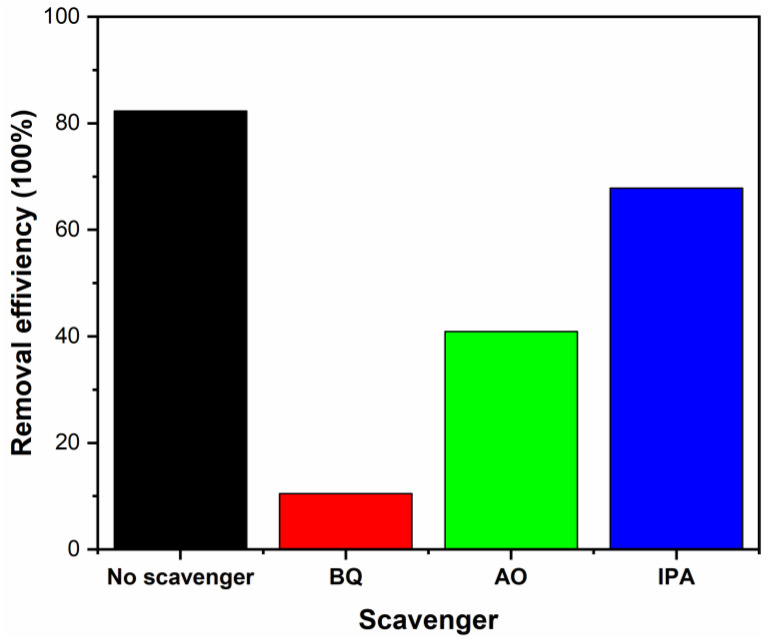
Degradation degrees of Ag@SnO_2_/TiO_2_ with different capture agents.

To determine the active species in photocatalytic reaction process, benzoquinone (BQ), ammonium oxalate (AO) and isopropanol (IPA) were added to capture the active groups, such as ·O_2_^−^, h^+^ and ·OH, during the TC photodegradation process by Ag@SnO_2_/TiO_2_ [51,52]. The results are shown in Figure 9. The degradation degree declines from 82.3 to 10.5% after adding BQ. The clearly decreased degradation degree indicates that ·O_2_^−^ radicals are the main active groups. The degradation degrees are 40.9 and 67.9% after adding AO and IPA, separately, implying that the holes and ·OH radicals play secondary roles in the degradation process.

### 3.8. The Degradation Mechanism

**Figure 10:**
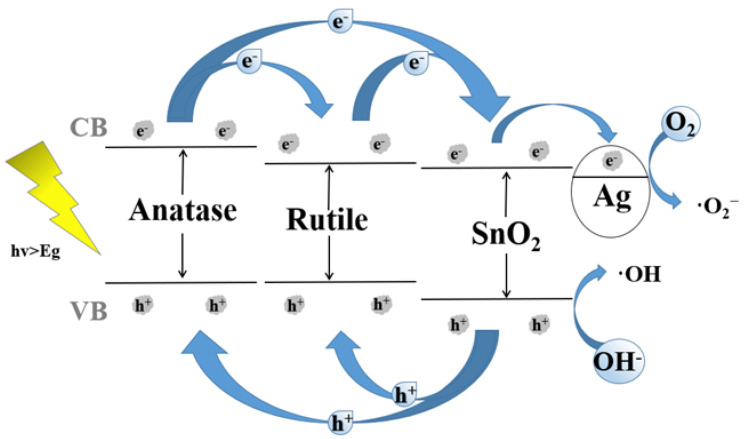
The energy band structure and the diagram of photogenerated charges transfer of Ag@SnO_2_/TiO_2_.

The energy band structure and the diagram of photogenerated charges transfer of Ag@SnO_2_/TiO_2_ are shown in Figure 10. When electrons are excited by photons, they will jump from the valence band to the conduction band, thus, generating photo-induced electrons. Since the conduction band and valence band of anatase and rutile are higher than SnO_2_, the photogenerated electrons in anatase and rutile will migrate to the conduction band of SnO_2_, and the holes in the valence band of SnO_2_ will transfer to the valence band of anatase and rutile.

The SnO_2_/anatase/rutile three-phase coexistence structure is beneficial to accelerating the photogenerated charges transfer, improving the quantum utilization [42,53]. Moreover, when Ag particles deposit on the surface of SnO_2_/anatase/rutile, the photogenerated electrons will migrate to the surface of Ag particles, further advancing the separation of photo-induced charges [54,55]. The rapid transfer of photogenerated charges at the interfaces of Ag@SnO_2_/anatase/rutile composites is conducive to the formation of more radicals and the improvement of the photocatalytic efficiency [34].

**Figure 11:**
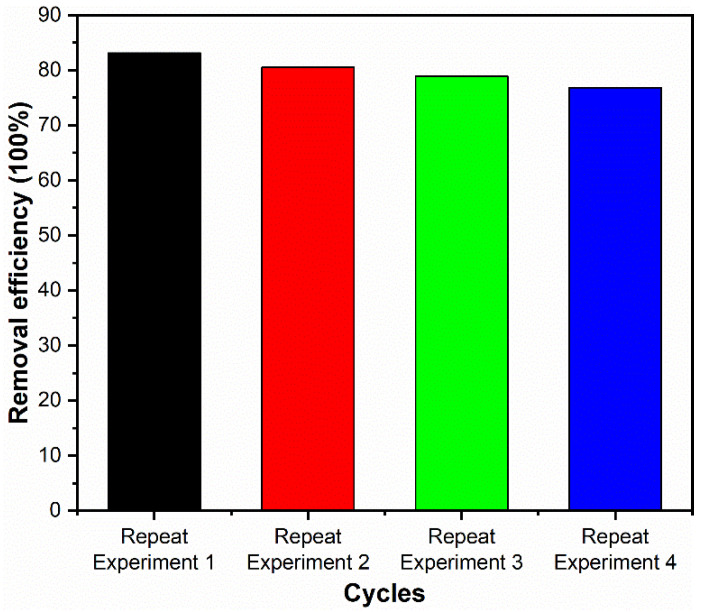
The cyclability of the Ag@SnO_2_/TiO_2_ photocatalyst for TC degradation.

The results of the cyclability of photocatalyst for TC degradation are shown in Figure 11. As the number of cycles increases, the degradation degree of TC decreases marginally. After four cycles, the degradation degree of TC by the Ag@SnO_2_/TiO_2_ composite photocatalyst decreases from 83.1 to 76.8%. The slight decrease in the degradation degree indicates that the composite photocatalyst shows relatively high reusability.

**Figure 12:**
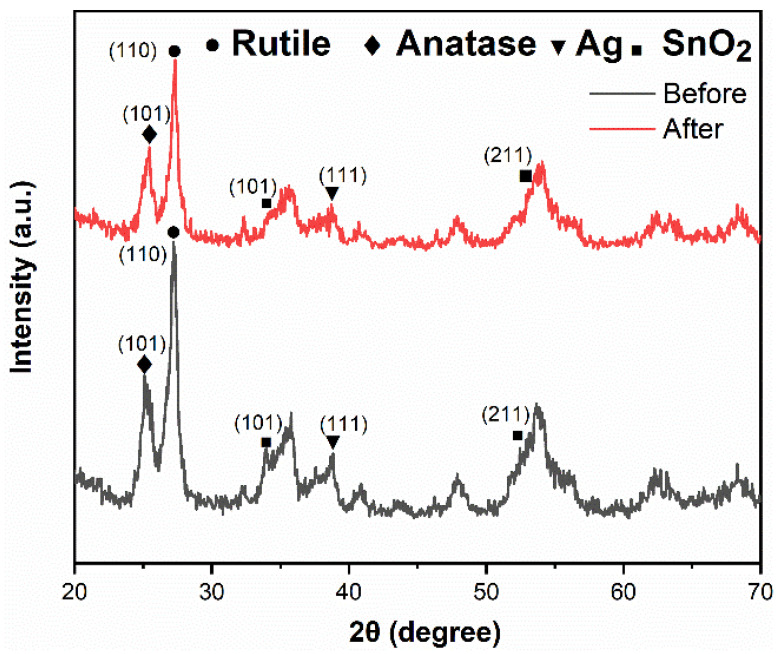
XRD patterns of Ag@SnO_2_/TiO_2_ photocatalyst before and after the photocatalytic experiment.

The XRD pattern of the Ag@SnO_2_/TiO_2_ composite photocatalyst after four cycles is shown in Figure 12. Compared with the initial sample, the positions of the diffraction peak do not change; however, the peak intensity decreases slightly. During the photocatalytic experiment, a very small amount of undegraded TC molecules may remain on the surface of photocatalyst, which decreases the diffraction peak intensity. On the other hand, the undegraded TC molecules occupy the active sites, resulting in a weak photocatalytic activity decrease [52,56,57]. This is also consistent with the repeated use experimental results.

## 4. Conclusions

We prepared anatase/rutile mixed crystal TiO_2_, Ag@TiO_2_, SnO_2_/TiO_2_, Ag decoration and SnO_2_ coupling modified TiO_2_ and constructed a Ag@SnO_2_/TiO_2_ composite photocatalytic material. XRD, SEM, TEM, XPS, PL, DRS and BET characterization and photocatalysis experiments were carried out on the samples. Ag exists in the form of 0 valence and Sn in the form of +4 valence. The results of the photocatalysis experiments showed that Ag decoration and SnO_2_ coupling enhanced the performance of anatase/rutile mixed crystal TiO_2_.

When Ag decoration and SnO_2_ coupling modification were implemented simultaneously, they had a synergistic effect on improving the charge separation and specific surface area, thus, exhibiting the highest photocatalytic activity. The first order reaction rate constant k of Ag@SnO_2_/TiO_2_ sample was 0.057 min^−1^, which was 1.7-times higher than that of pure TiO_2_ (0.034 min^−1^).

## Data Availability

Data is contained within the article.
